# Sodium-Glucose Co-Transporter 2 Inhibitors Correct Metabolic Maladaptation of Proximal Tubular Epithelial Cells in High-Glucose Conditions

**DOI:** 10.3390/ijms21207676

**Published:** 2020-10-16

**Authors:** Kohsuke Shirakawa, Motoaki Sano

**Affiliations:** 1 Department of Cardiovascular Medicine, Graduate School of Medicine, Juntendo University, Bunkyo-ku, Tokyo 113-8431, Japan; shirakawa19840905@gmail.com; 2 Department of Cardiology, Keio University School of Medicine, Shinjuku-ku, Tokyo 160-8582, Japan

**Keywords:** osteopontin, proximal tubular epithelial cells, SGLT2, GLUT2, 2-deoxy-D-glucose, myo-inositol oxygenase

## Abstract

Glucose filtered in the glomerulus is actively reabsorbed by sodium-glucose co-transporter 2 (SGLT2) in proximal tubular epithelial cells (PTEC) and passively returned to the blood via glucose transporter 2 (GLUT2). Healthy PTEC rely primarily on fatty acid beta-oxidation (FAO) for energy. In phase III trials, SGLT2 inhibitors improved outcomes in diabetic kidney disease (DKD). Tubulointerstitial renal fibrosis due to altered metabolic reprogramming of PTEC might be at the root of the pathogenesis of DKD. Here, we investigated the molecular mechanism of SGLT2 inhibitors’ renoprotective effect by examining transcriptional activity of *Spp1*, which encodes osteopontin, a key mediator of tubulointerstitial renal fibrosis. With primary cultured PTEC from *Spp1*-enhanced green fluorescent protein knock-in mice, we proved that in high-glucose conditions, increased SGLT2- and GLUT-mediated glucose uptake is causatively involved in aberrant activation of the glycolytic pathway in PTEC, thereby increasing mitochondrial reactive oxygen species (ROS) formation and transcriptional activation of *Spp1*. FAO activation did not play a direct role in these processes, but elevated expression of a tubular-specific enzyme, myo-inositol oxygenase, was at least partly involved. Notably, canagliflozin blocked overexpression of myo-inositol oxygenase. In conclusion, SGLT2 inhibitors exerted renoprotective effects by inhibiting aberrant glycolytic metabolism and mitochondrial ROS formation in PTEC in high-glucose conditions.

## 1. Introduction

Damage to proximal tubular epithelial cells (PTEC), which account for 90% of the renal cortex, has attracted attention as a final common pathway for a wide variety of kidney diseases, including diabetic kidney disease (DKD) [1]. Clinical trials have shown that drugs that inhibit sodium-glucose cotransporter 2 (SGLT2), which is expressed in PTEC, improve the prognosis of DKD, lending credence to this theory [2,3,4,5,6,7,8,9,10,11].

PTEC require an enormous amount of energy to transport a variety of substances [12]. Approximately 65% of electrolytes and 100% of glucose and amino acids filtered by the glomerulus are reabsorbed by the proximal tubules. PTEC are rich in mitochondria and produce adenosine triphosphate (ATP), mainly through the beta-oxidation of fatty acids [12]. The proximal tubule is also the second most important organ after the liver for gluconeogenesis, which occurs after its cells have produced ammonia from glutamine [12], but its glycolytic system is underdeveloped.

In the first half of the proximal tubule (S1-S2 segment), SGLT2 in the apical membrane (luminal side of the tubule) reabsorbs 90% of the filtered glucose as part of a high-capacity system [13]. SGLT2-mediated reabsorption of glucose couples with Na^+^/K^+^-ATPase in PTEC on the basolateral membrane (vascular lumen side) to create the concentration gradient of sodium ions inside and outside the cells [14]. In diabetic patients, Na^+^/K^+^ pump activity and glucose reabsorption via SGLT2 are enhanced [14] and, not surprisingly, renal cortical oxygen consumption is increased [15]. Interestingly, a study in a rat model of diabetes showed that enhanced renal cortical oxygen consumption and the resulting reduction in the tissue partial pressure of oxygen were reversed by inhibiting SGLT2 [16].

Metabolic reprogramming, as evidenced by maladaptive changes in metabolism, can lead to tissue dysfunction. Recently, a study reported that in pathological conditions metabolic flux in the glycolytic system and expression of glycolytic enzymes in the proximal tubules were increased [17,18,19]. In addition, in diabetes, myo-inositol intracellular concentrations were reduced by the upregulation of the myo-inositol-degrading enzyme myo-inositol oxygenase (MIOX), a tubular-specific enzyme, and the glucuronate xylulose pathway was activated [20,21,22]. The activation of myo-inositol catabolism induces mitochondrial fragmentation and depolarization but inhibits autophagic removal of damaged mitochondria, resulting in accumulation of dysfunctional mitochondria; these dysfunctional mitochondria generate excessive reactive oxygen species (ROS) and initiate apoptotic cascade, leading to tubular injury [23]. Tubular injury can lead to tubulointerstitial fibrosis, which contributes to kidney dysfunction and causes end-stage kidney failure, the net result of abnormalities in many metabolic and signaling pathways. Osteopontin (OPN) plays a critical role in interstitial fibrosis [24,25]. In both humans and mice, OPN is expressed in kidneys in the steady state, mainly in the ascending limb of loop of Henle and distal tubules [26]. However, in pathological conditions, such as ischemia-reperfusion injury, OPN is highly expressed in the proximal tubules [27,28]. OPN expression in the proximal tubules has also been reported to be markedly upregulated in db/db mice (a mouse model of diabetes) and in mice with streptozocin-induced diabetes [29].

In this study, in order to gain insight into the mechanism of action of SGLT2 inhibitors, we examined their effects on metabolic remodeling induced by high-glucose conditions, mitochondrial oxidative stress, and the production of profibrogenic OPN in primary cultured PTEC. Our primary conclusion was that SGLT2 inhibitors exert renoprotective effects by inhibiting aberrant glycolytic metabolism and mitochondrial ROS formation in PTEC in high-glucose conditions.

## 2. Results

### 2.1. High-Glucose Conditions Induce Spp1 Transcriptional Activity in PTEC

**Figure 1:**
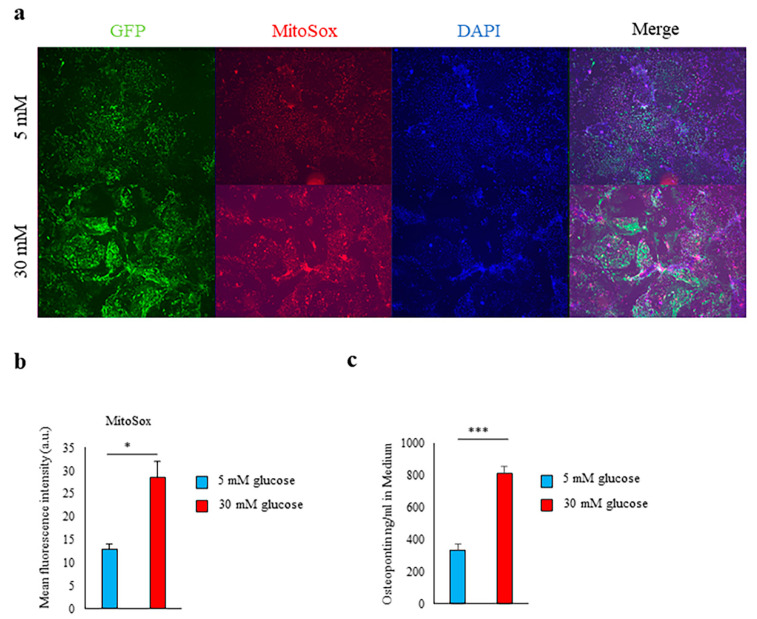
Hyperglycemia induced *Spp1* transcriptional activity in renal proximal tubular epithelial cells. (**a–c**) Primary culture of proximal tubular epithelial cells (PTEC) derived from enhanced green fluorescent protein (EGFP)-*Spp1* knock-in reporter mice. PTEC were cultured in 5 mM or 30 mM glucose conditions for 7 days. (**a**) Microscopic analysis of fluorescence of 4ʹ,6-diamidino-2-phenylindole (DAPI) and MitoSOX staining and of EGFP-*Spp1* expression. (**b**) Mean fluorescence intensity of MitoSox in PTEC (*n* = 3 per group). (**c**) Osteopontin (OPN) in the culture supernatants was assessed by enzyme-linked immunosorbent assay (ELISA; *n* = 6 per group). Data are shown as means, and error bars depict SEM. * *p* < 0.05, *** *p* < 0.001.

We established a primary culture of PTEC from enhanced green fluorescent protein (EGFP) knock-in mice for *Spp1*, which encodes the OPN gene. The PTEC were cultured at glucose concentrations of 5 mM (90 mg/dL; normoglycemic equivalent) and 30 mM (540 mg/dL; hyperglycemic equivalent) for 7 days. Although little *Spp1* transcriptional activity occurred in the PTEC cultured at 5 mM, the transcriptional activity of *Spp1*, production of OPN, and formation of mitochondrial ROS were markedly increased in the PTEC cultured at 30 mM (Figure 1a–c).

### 2.2. Induction of Spp1 Transcriptional Activity in PTEC by High Glucose Was Reversible

**Figure 2:**
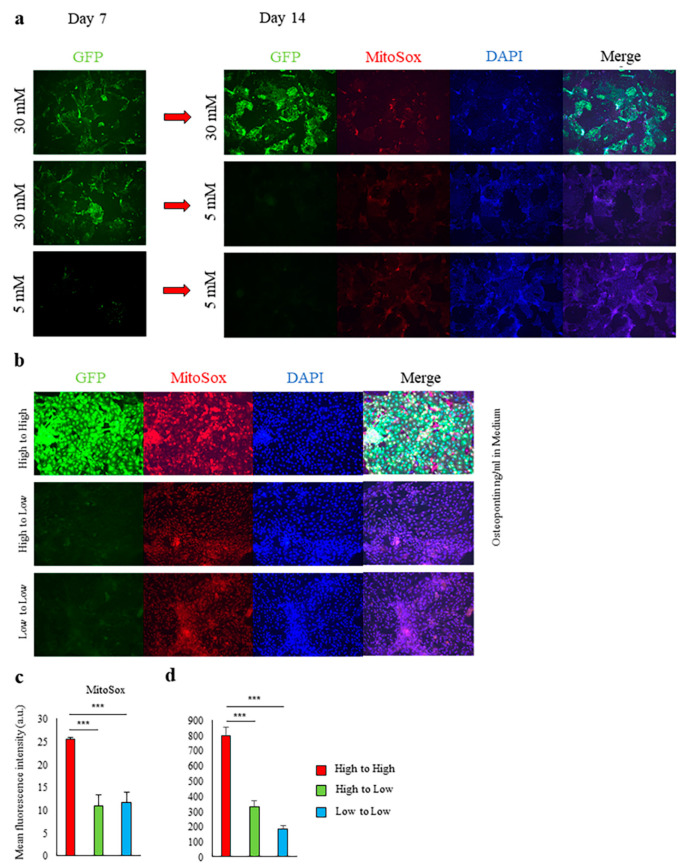
Glycemic control reversibly regulated *Spp1* transcriptional activity of renal proximal tubular epithelial cells. (**a**,**b**) Primary culture of proximal tubular epithelial cells (PTEC) derived from enhanced green fluorescent protein (EGFP)-*Spp1* knock-in reporter mice. PTEC were cultured in 5 mM (“low”) or 30 mM (“high”) glucose conditions for 7 days, then cultured in the indicated conditions for another 7 days. Microscopic analysis of fluorescence of 4ʹ,6-diamidino-2-phenylindole (DAPI) and MitoSOX staining and of EGFP-*Spp1* expression. (**c**) Mean fluorescence intensity of MitoSox in PTEC (*n* = 4–6 per group). (**d**) Osteopontin (OPN) in the culture supernatants was assessed by enzyme-linked immunosorbent assay (*n* = 6 per group). Data are shown as means, and error bars depict SEM. *** *p* < 0.001.

*Spp1* transcriptional activity and mitochondrial ROS formation were markedly decreased when PTEC from *Spp1* EGFP knock-in mice were cultured in 30 mM high-glucose conditions for 7 days and then cultured in 5 mM low-glucose conditions for an additional 7 days (Figure 2a–d).

### 2.3. Fatty Acid Beta-Oxidation Was Not Involved in Spp1 Transcriptional Activation in High-Glucose Conditions

**Figure 3:**
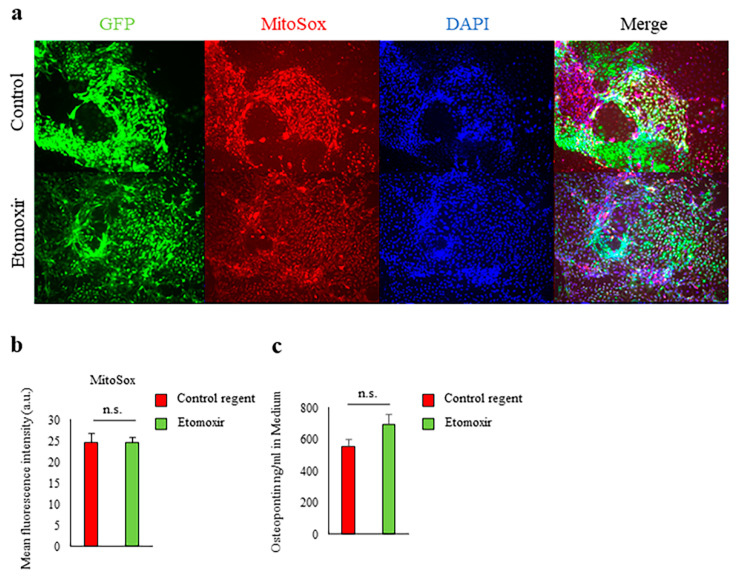
Fatty acid beta-oxidation was not involved in *Spp1* transcriptional activation in high-glucose conditions. (**a**) Primary culture of proximal tubular epithelial cells (PTEC) derived from enhanced green fluorescent protein (EGFP)-*Spp1* knock-in reporter mice. PTEC were cultured in 30 mM glucose conditions for 7 days with etomoxir or control regent. Microscopic analysis of fluorescence of 4ʹ,6-diamidino-2-phenylindole (DAPI) and MitoSOX staining and of EGFP-*Spp1* expression. (**b**) Mean fluorescence intensity of MitoSox in PTEC (*n* = 6 per group). (**c**) Osteopontin (OPN) in the culture supernatants was assessed by enzyme-linked immunosorbent assay (*n* = 6 per group). n.s., not significant. Data are shown as means, and error bars depict SEM.

PTEC from *Spp1* EGFP knock-in mice were cultured in 30 mM glucose in the presence or absence of the carnitine palmitoyltransferase 1 (CPT-1) inhibitor etomoxir for 7 days. Inhibition of fatty acid beta-oxidation by etomoxir had no effect on mitochondrial ROS formation, *Spp1* transcriptional activation, or OPN production (Figure 3a–c).

### 2.4. Glucose Transporter and SGLT2-Mediated Glucose Influx and Activation of the Glycolytic Pathway Were Involved in Spp1 Transcriptional Activation in the High-Glucose Conditions

**Figure 4:**
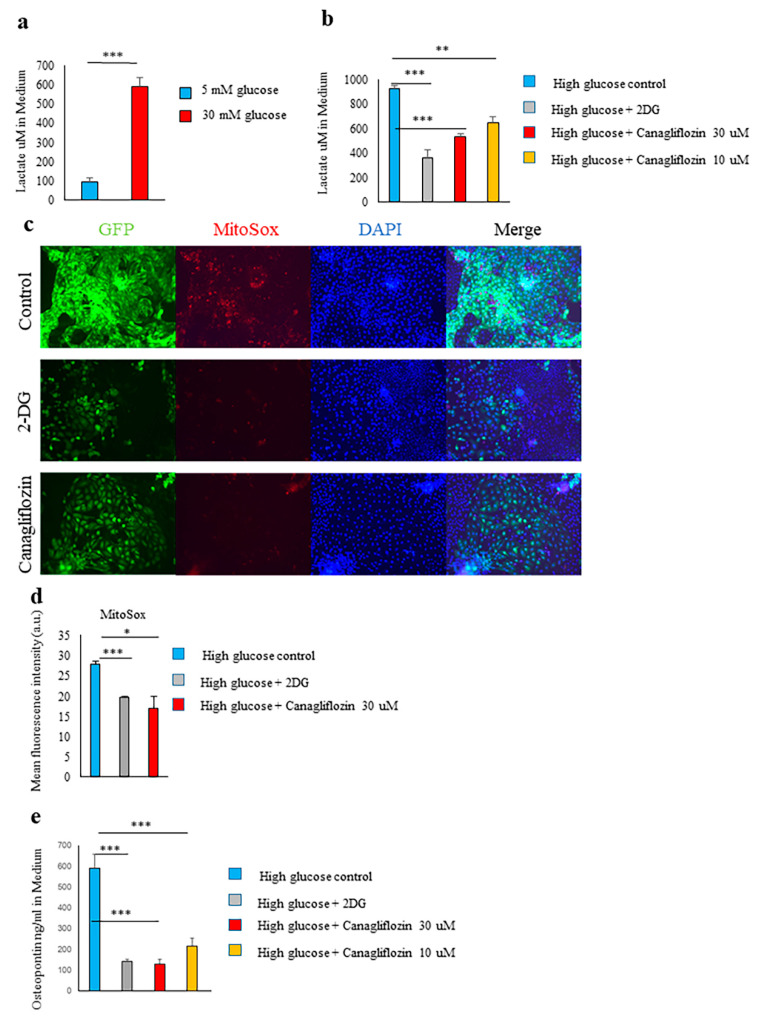
Canagliflozin ameliorated the upregulation of *Spp1* transcriptional activity. (**a**) Primary culture of proximal tubular epithelial cells (PTEC) derived from enhanced green fluorescent protein (EGFP)-*Spp1* knock-in reporter mice. PTEC were cultured in 5 mM (“low”) or 30 mM (“high”) glucose conditions for 7 days. Lactate in the culture supernatants was assessed by enzyme-linked immunosorbent assay (ELISA; *n* = 6 per group). (**b**–**d**) Primary culture of PTEC derived from EGFP-*Spp1* knock-in reporter mice. PTEC were cultured in 30 mM glucose conditions for 7 days with 50 mM 2-deoxy-D-glucose (2-DG), 30-µM canagliflozin, 10-µM canagliflozin, or control regent. (**b**) Lactate in the culture supernatants was assessed by ELISA (*n* = 6 per group). (**c**) Analysis of fluorescence microscopy showed 4ʹ,6-diamidino-2-phenylindole (DAPI) and MitoSOX staining and of EGFP-*Spp1* expression. (**d**) Mean fluorescence intensity of MitoSox in PTEC (*n* = 3–5 per group). (**e**) Osteopontin (OPN) in the culture supernatants was assessed by ELISA (*n* = 6 per group). * *p* < 0.05, ** *p* < 0.01, *** *p* < 0.001. Data are shown as means, and error bars depict SEM.

When PTEC were cultured in high-glucose conditions, we found that lactate production was markedly higher than in low-glucose conditions (Figure 4a). These results suggest that the aerobic glycolytic system was enhanced in the PTEC under high-glucose conditions.

Intracellular uptake of glucose via SGLT2 and glucose transporter 2 (GLUT2) is known to be enhanced in the tubules of diabetic patients, rodent models of diabetes, and in PTEC cultured in high-glucose conditions [30]. The glucose molecule 2-deoxy-D-glucose (2-DG) is a substrate for glucose transporters (GLUTs) but not a substrate for sodium-glucose cotransporters (SGLTs). 2-DG enters the cell through GLUTs and is phosphorylated by hexokinase, forming 2-DG-6-phosphate. Low intracellular levels of phosphatase cause 2-DG-6-phosphate to be trapped in the cell, where it is unable to undergo further metabolism. The resulting high intracellular levels of 2-DG-6-phosphate cause allosteric and competitive inhibition of hexokinase, thus inhibiting the glycolytic pathway [31]. In our study, inhibition of the intracellular influx of glucose via GLUT and of hexokinase by 2-DG strongly suppressed the lactate production, mitochondrial ROS formation, *Spp1* transcriptional activity, and OPN production induced by the high-glucose conditions, as did inhibition of SGLT2-mediated intracellular glucose uptake by canagliflozin (Figure 4a–e).

### 2.5. Canagliflozin and 2-DG Inhibited High Glucose-Induced MIOX Upregulation

Myo-inositol is synthesized from glucose-6-phosphate [32]. In high-glucose conditions, myo-inositol catabolism is known to be enhanced by myo-inositol oxygenase (MIOX) overexpression, which increases the oxidative stress on PTEC and promotes tubulointerstitial fibrosis [32].

**Figure 5:**
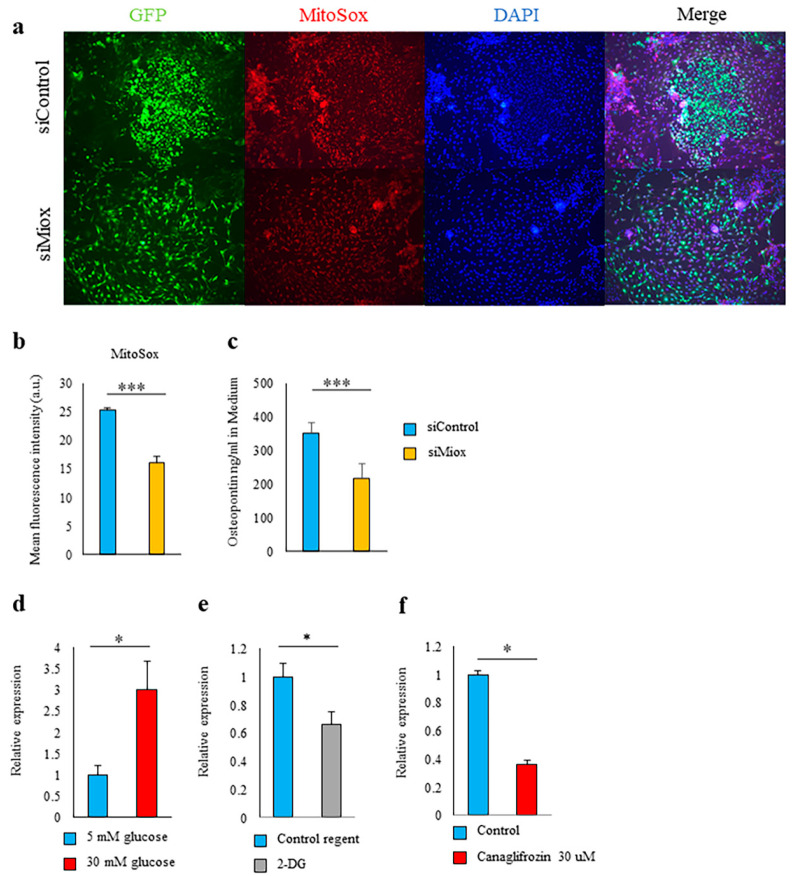
Upregulation of myo-inositol oxygenase (MIOX) regulated the *Spp1* transcriptional activity in the proximal renal tubular cells. (**a**–**c**) Proximal tubular epithelial cells (PTEC) were cultured for 7 days with small interfering (si)Control or siMIOX. (**a**) Microscopic analysis of fluorescence of 4ʹ,6-diamidino-2-phenylindole (DAPI) and MitoSOX staining and of EGFP-*Spp1* expression. (**b**) Mean fluorescence intensity of MitoSox in PTEC (*n* = 4–6 per group). (**c**) Osteopontin (OPN) in the culture supernatants was assessed by enzyme-linked immunosorbent assay (*n* = 6 per group). (**d**–**f**) The mRNA expression levels of MIOX were quantified in PTEC cultured in the indicated conditions. * *p* < 0.05, *** *p* < 0.001. Data are shown as means, and error bars depict SEM.

In our primary cultured PTEC, we could confirm that the expression of MIOX was upregulated in high-glucose conditions. Knocking down MIOX with small interfering RNA (siRNA) reduced mitochondrial oxidative stress and suppressed *Spp1* transcriptional activity and OPN production (Figure 5a–d). Notably, the upregulation of MIOX in high-glucose conditions was inhibited by both 2-DG and canagliflozin (Figure 5e,f).

## 3. Discussion

Multiple theories have been proposed as to the mechanism by which SGLT2 inhibitors improve the prognosis of DKD [9,33,34]. Because maladaptive changes in cellular metabolism would lead to tissue dysfunction [17,18,19], we investigated the impact of canagliflozin on the altered metabolism of PTEC in high-glucose conditions. Our study showed that canagliflozin may exert a renoprotective effect by restoring the maladaptive changes in the metabolism of PTEC, in particular the aberrant glycolytic metabolism.

A systems approach with transcriptomics, metabolomics, and metabolic flux analysis in both 12- and 24-week-old db/db type 2 diabetic mice outlined an increase in glycolysis in the diabetic kidney cortex [19]. However, whether this metabolic reprogramming is adaptive or maladaptive has not been fully elucidated in terms of its pathophysiological significance. In high-glucose conditions, both SGLT2 and GLUT2 are upregulated in PTEC [35,36]. The influx of glucose from them flows into the glycolytic pathway, which is also activated. This process appears to exert a cytotoxic effect [23]. 2-DG blocks the glycolytic pathway itself by inhibiting hexokinase at the same time as it inhibits GLUT, thus more potently preventing the transcriptional activity of *Spp1* and oxidative stress. On the other hand, canagliflozin inhibits only SGLT2-mediated glucose influx into the cell, and thus its effect is naturally inferior to that of 2-DG. However, when administered systemically, the side effects of 2-DG, which inhibits the glycolytic system itself, are too strong. Inhibitors of SGLT2 that are exclusively expressed in PTEC have an excellent benefit–risk balance. In fact, SGLT2 inhibitors have been shown to be well tolerated in patients with heart failure and chronic kidney disease (CKD), as demonstrated by extremely high participant retention rates in phase III clinical trials [37,38].

Intracellular concentrations of myo-inositol are determined by cellular uptake through inositol transporters, endogenous synthesis via glucose-6-phosphate, and degradation via the glucuronate xylulose pathway. MIOX, a tubular-specific enzyme, modulates redox imbalance and apoptosis in tubular cells in diabetes, resulting in tubulointerstitial fibrosis [20,22]. The expression of MIOX was also elevated in our culture system in high-glucose conditions. Suppression of MIOX expression with siRNA significantly suppressed the transcriptional activity of *Spp1* and the mitochondrial ROS production induced by high-glucose conditions. Importantly, the elevated expression of MIOX by high-glucose conditions was suppressed by canagliflozin and 2-DG. These results suggest that enhanced myo-inositol catabolism may be involved in at least some of the glucotoxicity associated with an increased intracellular glucose influx in PTEC.

High serum concentrations of OPN have been reported to be negatively correlated with the estimated glomerular filtration rate in patients with chronic kidney disease [39], and serum OPN levels have been reported to be elevated in patients with heart failure [40]. Furthermore, serum OPN concentrations are a predictor of ventricular tachycardia and ventricular fibrillation in patients with heart failure [41]. Although the molecular mechanism of cardiorenal syndrome remains unclear, our results suggest canagliflozin suppresses the glucose load on pathological PTEC, which have shifted the metabolic pathway to the glycolytic system, and thus suppresses the production of OPN, thereby reducing the occurrence of cardiorenal events.

In conclusion, SGLT2 inhibitors may exert a renoprotective effect by correcting maladaptive changes in metabolism of PTEC in a hyperglycemic environment.

## 4. Materials and Methods

### 4.1. Animal Care

C57BL/6 (B6) mice were purchased from Clea Japan. EGFP-*Spp1* knock-in reporter mice were kindly provided by Nagahiro Minato (Kyoto University, Japan). All mice were bred according to husbandry guidelines for C57BL/6 mice. We used 8- to 10-week-old male mice. The study conformed with the “Guide for the Care and Use of Laboratory Animals” published by the U.S. National Institute of Health (NIH publication no. 85-23, revised 1996), and the study protocol was approved by the Institutional Animal Care and Use Committee at the Keio University School of Medicine (Ethical approval code: 17057).

### 4.2. Proximal Tubule Isolation and Primary Culture

We isolated proximal tubular cells from male EGFP-*Spp1* knock-in reporter mice by previously described methods [42] with some modifications. Mice were deeply anesthetized and intracardially perfused with 40 mL of ice-cold phosphate-buffered saline (PBS) to exclude blood cells. The kidneys were removed and immediately placed into ice-cold PBS, and the cortex was carefully dissected away from the medulla. The cortex was finely minced and transferred into pre-warmed Hank’s balanced salt solution (HBSS) with DNsae (20 μg/mL), collagenase (1.4 mg/mL), and trypsin inhibitor (0.033 mg/mL), and gently mixed at 37 °C for 30 min. After digestion, the supernatant was sieved through an 80-µm nylon sieve and gently washed several times in HBSS; cellular debris and glomeruli were discarded. The material on the top of the sieve was collected and centrifuged at 300× *g* for 5 min. The supernatant was decanted, and the material was resuspended in DMEM/F12 without phenol red (containing 5 mM or 30 mM D-glucose, 1% FBS, 15 mM HEPES, 1× insulin/transferrin/selenium solution, 100 IU/mL penicillin, 100 µg/mL streptomycin, and 50 nM hydrocortisone) and seeded into 24-well gelatin-coated plates (Thermo Fisher Scientific, Waltham, MA, USA). Cells were cultivated at 37 °C with 5% CO_2_ at 80% confluence and were treated with or without 10 μM or 30 μM canagliflozin (Mitsubishi Tanabe Pharma Corporation, Tokyo, Japan), 50 mM 2-DG, and 40 μM etomoxir or control regent for 48 h.

### 4.3. Primary Culture of PTEC with Varying Concentrations of Glucose in the Culture Medium

PTEC derived from EGFP-*Spp1* knock-in reporter mice were cultured in 5 mM (“low”) or 30 mM (“high”) glucose conditions for 7 days, then cultured in 5 mM (“low”) or 30 mM (“high”) glucose conditions for another 7 days (“high to high”, “high to low”, and “low to low”).

### 4.4. Genetic Knock-Down and Expression of PTEC

For knock-down experiments, we transfected PTEC with MIOX or control siRNA (Sigma-Aldrich, St. Louis, MO, USA) by using Lipofectamine 3000 (Invitrogen, Waltham, MA, USA) according to the manufacturer’s protocol.

### 4.5. Detection of ROS in Primary Culture of PTEC

To examine mitochondrial ROS levels, we loaded cultured PTEC with 5 μM MitoSOX (Life Technologies, Waltham, MA, USA) for 10 min at 37 °C, according to the manufacturer’s instructions. The nucleus was stained with Hoechst 33,342 for 10 min at 37 °C. Results were examined with software (BZ-H1C; Keyence, Osaka, Japan), and micrographs were taken from each section at 20 magnification with a digital camera (BIOREVO; Keyence, Osaka, Japan).

### 4.6. Enzyme-Linked Immunosorbent Assay (ELISA)

The levels of OPN (R&D Systems, Minneapolis, MN, USA) in supernatants was determined by ELISA according to the manufacturers’ instructions.

### 4.7. Lactate Assay

Levels of lactate in supernatants were determined by Lactate Assay Kit-WST (DOJINDO, Kumamoto, Japan) according to the manufacturer’s instructions.

### 4.8. Quantitative Real-Time Polymerase Chain Reaction

Total RNA samples from PTEC were prepared with the RNeasy Mini Kit (Qiagen, Hilden, Germany), according to the manufacturer’s instructions. The First-Strand cDNA Synthesis Kit (Invitrogen, Waltham, MA, USA) was used for cDNA synthesis. Quantitative real-time polymerase chain reaction was performed with the ViiA 7 Real-Time PCR System (Applied Biosystems, Waltham, MA, USA). *18S* was used as an endogenous control to normalize for differences in the amount of total RNA in each sample. All values are expressed as fold increase or decrease relative to the expression of *18S*. Primer sequences for genes were as follows: *Miox*, 5′-CCCTTCCCTGGTCTATCGAC-3′ and 5′-GTGGTAAAGACACGATCCAGC-3′; *18S*, 5′-CGAACGTCTGCCCTATCAACTT-3′ and 5′-ACCCGTGGTCACCATGGT-3′.

### 4.9. Statistical Analysis

All values are presented as means (SEM). The statistical significance of differences between two groups was determined by two-sided unpaired Student’s *t*-tests. *p* < 0.05 was considered statistically significant.

## Abbreviations

ATP	Adenosine triphosphate
EGFP	Enhanced green fluorescent protein
DKD	Diabetic kidney disease
GLUT2	Glucose transporter 2
*Miox*	Myo-inositol-degrading enzyme myo-inositol oxygenase
OPN	Osteopontin
PTEC	Proximal tubular epithelial cells
SGLT2	Sodium-glucose cotransporter 2
siRNA	Small interfering RNA
ROS	Reactive oxygen species
